# RETRACTION: Meta‐Analysis Comparing Different Ultrasound Detection Methods to Accurately Assess Wound Healing and Scar Formation after Caesarean Section

**DOI:** 10.1111/iwj.70161

**Published:** 2024-12-15

## Abstract

**Retraction:** MiaoJ.‐J. , LuoY.‐Y. , WangC.‐Y. , LiH.‐X. , and YuH.‐X. , “Meta‐Analysis Comparing Different Ultrasound Detection Methods to Accurately Assess Wound Healing and Scar Formation after Caesarean Section,” International Wound Journal 21, no. 4 (2024): e14837, 10.1111/iwj.14837.38629613 PMC11022302

The above article, published online on 17 April 2024, in Wiley Online Library (http://onlinelibrary.wiley.com/), has been retracted by agreement between the journal Editor in Chief, Professor Keith Harding; and John Wiley & Sons Ltd. Following an investigation by the publisher, all parties have concluded that this article was accepted solely on the basis of a compromised peer review process. The editors have therefore decided to retract the article. The authors did not respond to our notice regarding the retraction.

**Retraction:** J.‐J. Miao , Y.‐Y. Luo , C.‐Y. Wang , H.‐X. Li , and H.‐X. Yu , “Meta‐Analysis Comparing Different Ultrasound Detection Methods to Accurately Assess Wound Healing and Scar Formation after Caesarean Section,” International Wound Journal 21, no. 4 (2024): e14837, 10.1111/iwj.14837.38629613 PMC11022302

The above article, published online on 17 April 2024, in Wiley Online Library (http://onlinelibrary.wiley.com/), has been retracted by agreement between the journal Editor in Chief, Professor Keith Harding; and John Wiley & Sons Ltd. Following an investigation by the publisher, all parties have concluded that this article was accepted solely on the basis of a compromised peer review process. The editors have therefore decided to retract the article. The authors did not respond to our notice regarding the retraction.

